# Mouse DCUN1D1 (SCCRO) is required for spermatogenetic individualization

**DOI:** 10.1371/journal.pone.0209995

**Published:** 2019-01-17

**Authors:** Guochang Huang, Andrew J. Kaufman, Russell J. H. Ryan, Yevgeniy Romin, Laryssa Huryn, Sarina Bains, Katia Manova-Todorova, Patricia L. Morris, Gary R. Hunnicutt, Carrie A. Adelman, John H. J. Petrini, Y. Ramanathan, Bhuvanesh Singh

**Affiliations:** 1 Department of Surgery, Laboratory of Epithelial Cancer Biology, Memorial Sloan Kettering Cancer Center, New York, New York, United States of America; 2 Molecular Cytology Core Facility, Memorial Sloan Kettering Cancer Center, New York, New York, United States of America; 3 Population Council and The Rockefeller University, New York, New York, United States of America; 4 Department of Molecular Biology, Memorial Sloan Kettering Cancer Center, New York, New York, United States of America; University Hospital of Münster, GERMANY

## Abstract

Squamous cell carcinoma–related oncogene (*SCCRO*, also known as *DCUN1D1*) is a component of the E3 for neddylation. As such, DCUN1D1 regulates the neddylation of cullin family members. Targeted inactivation of *DCUN1D1* in mice results in male-specific infertility. Infertility in *DCUN1D1*^*-/-*^ mice is secondary to primary defects in spermatogenesis. Time-dam experiments mapped the onset of the defect in spermatogenesis to 5.5 to 6 weeks of age, which temporally corresponds to defects in spermiogenesis. Although the first round of spermatogenesis progressed normally, the number of spermatozoa released into the seminiferous lumen and epididymis of *DCUN1D1*^*-/-*^ mice was significantly reduced. Spermatozoa in *DCUN1D1*^*-/-*^ mice had multiple abnormalities, including globozoospermia, macrocephaly, and multiple flagella. Many of the malformed spermatozoa in *DCUN1D1*^*-/-*^ mice were multinucleated, with supernumerary and malpositioned centrioles, suggesting a defect in the resolution of intercellular bridges. The onset of the defect in spermatogenesis in *DCUN1D1*^*-/-*^ mice corresponds to an increase in DCUN1D1 expression observed during normal spermatogenesis. Moreover, consistent with its known function as a component of the E3 in neddylation, the pattern of DCUN1D1 expression temporally correlates with an increase in the neddylated cullin fraction and stage-specific increases in the total ubiquitinated protein pool in wild-type mice. Levels of neddylated Cul3 were decreased in *DCUN1D1*^*-/-*^ mice, and ubiquitinated proteins did not accumulate during the stages in which DCUN1D1 expression peaks during spermatogenesis in wild-type mice. Combined, these findings suggest that *DCUN1D1*^*-/-*^ mice fail to release mature spermatozoa into the seminiferous lumen, possibly due to unresolved intercellular bridges. Furthermore, the effects of DCUN1D1 on spermatogenesis likely involve its regulation of cullin-RING-ligase (CRL)–type ubiquitin E3 activity during spermiogenesis through its role in promoting Cul3 neddylation. The specific CRLs required for spermiogenesis and their protein targets require identification.

## Introduction

Cullin-RING–based E3 ubiquitin ligases (CRLs), the largest class of mammalian ubiquitination E3s [1], are specified by more than 600 human genes and are involved in the control of many cellular processes [2]. The assembly and activity of RING E3s are regulated in large part by neddylation, the posttranslational modification of cullins by Nedd8 [3]. Neddylation, a process analogous to ubiquitination in which a tripartite cascade results in covalent modification of the cullin family of proteins by the ubiquitin-like protein Nedd8, is regulated by the activity of the E3 for neddylation [4–6]. Despite the critical importance of neddylation, the specifics of how this process is regulated and the CRLs and proteins affected by it remain poorly understood.

Recently, we and others identified squamous cell carcinoma–related oncogene (*SCCRO*, also known as *DCUN1D1)* and showed that it functions as a regulatory component of the neddylation E3 [7–11]. DCUN1D1 promotes neddylation in three ways: (1) promoting nuclear translocation of cullin-ROC1 complexes, (2) enhancing recruitment of E2~Nedd8 (Ubc12~Nedd8) thioester to the complex, and (3) optimizing the orientation of proteins in the complex to allow efficient transfer of Nedd8 from the E2 to cullins. Although mutations in the core components of this pathway (including Roc1, Cul1, Cul3, and Uba3) result in early embryonic lethality in mice [12–16], *DCUN1D1*-knockout mice are viable, likely owing to compensation from paralogs present exclusively in higher organisms [17]. Phenotypic assessment of *DCUN1D1*^*-/-*^ mice identified no gross abnormalities in all major organs, with the exception of the testis. Loss of DCUN1D1 in the testis results in male-specific infertility in *DCUN1D1*^*-/-*^ mice.

In this study, we sought to determine the basis of infertility in male *DCUN1D1*^*-/-*^ mice. We mapped the fertility defect to defects in spermatogenesis that are likely caused by unresolved intercellular bridges during spermiogenesis. Furthermore, we showed that decreased cullin neddylation and CRL-promoted ubiquitination in *DCUN1D1*^-/-^ mice temporally correlate with defective spermiogenesis.

## Materials and methods

### Gene targeting and generation of homozygous mice

Male chimeric mice were generated from the embryonic stem (ES) cell line RRR261, obtained from BayGenomics [18]. The RRR261 ES cell line was generated using the retroviral gene trap vector pGT01xf, which contains the intron from the engrailed 2 gene upstream of the gene encoding the β-galactosidase/neomycin-resistance fusion protein (https://www.mmrrc.org). ES cell clones with proper entrapment of *DCUN1D1* were confirmed by the use of 5’ rapid amplification of cDNA ends (RACE) RT-PCR, using a 5’ RACE system (Invitrogen), in accordance with the manufacturer’s protocol and as described elsewhere (http://www.sanger.ac.uk/PostGenomics/genetrap/protocols). We used a primer for cDNA synthesis (5’-TAATGGGATAGGTTACG-3’) and primers for the nested PCR (5’-AGTATCGGCCTCAGGAAGATCG-3’ and 5’-ATTCAGGCTGCGCAACTGTTGGG-3’).

The germline transmission of injected ES cells was confirmed by 5’ RACE RT-PCR of resultant litters, under the same conditions as above. Heterozygous mice were screened using PCR with forward and reverse primers internal to the β-galactosidase/neomycin-resistance gene (forward: 5’-TTATCGATGAGCGTGGTGGTTATGC-3’; reverse: 5’-GCGCGTACATCGGGCAAATAATATC-3’), creating a 680-bp product.

To identify the site of vector integration, PCR with successive forward primers specific to *DCUN1D1* intron 1 were used with the β-galactosidase/neomycin-resistance–specific reverse primer mentioned above. The PCR product was sequenced to confirm the site of integration. A multiplex PCR genotyping protocol was designed using a common forward primer from intron 1 upstream of the insertion (5’-TTTGTGCACTTGGGCTCATGAAGG-3’) and two different reverse primers: one in intron 1 downstream from the 3’ end of the vector (5’-TTGTCAATCCCCCAGTGTTAAG-3’) and the other a vector-specific primer (5’-TTCACTGAGTCTCTGGCATCTC-3’). Products of 1074 bp and 963 bp—wild-type and mutant, respectively—were visualized on 1% agarose gel. Southern blot analysis of DNA following digestion of genomic DNA with PvuII confirmed the PCR genotyping results. An external DNA probe approximately 360 bp in length was amplified from intron 1 upstream of the vector insertion using the following forward and reverse primers: 5’-CGTGGCTAGCATAAAGTTTGAA and 5’-TAAAGGTTCTGCGTGTCGTAGGCA. The probe was labeled with ^32^P alpha–labeled deoxycytidine triphosphate.

### Mouse breeding and litter analysis

This study was carried out in strict accordance with the recommendations in the Guide for the Care and Use of Laboratory Animals of the National Institutes of Health, following federal regulations and policies governed by the Animal Welfare Act and the Health Research Extension Act of 1985. The animal care and use program within Memorial Sloan Kettering Cancer Center is overseen by the Research Animal Resource Center, under the direction of the Institutional Animal Care and Use Committee. The protocol was approved by Institutional Animal Care and Use Committee at our institution (Protocol Number: 08-09-021). Heterozygote male and female mice were back-crossed with C57BL/6J mice for 6 generations and then intercrossed to produce homozygous *DCUN1D1*^*-/-*^ mice. Pups were marked according to Institutional Animal Care and Use Committee protocols. Data on weight and length were recorded prospectively from the day of birth until adulthood. Humane endpoints were used in all aspects of this study. All researchers involved in this study had skills and training necessary to care for animals used in this research. All staff completed training required by our institution. Clinical care was also provided by a dedicated Veterinary Services section at our institution. The Veterinary Services section consists of a team of veterinary technicians and specially trained veterinarians. The Veterinary Services section was consulted and provided professional and technical services, clinical care, and preventive medicine to assure animal health and humane care during the course of this study. Perinatal lethality was observed while breeding DCUN1D1-knockout mice. All pups were subjected to behavioral observations twice a day, including movement outside and inside nest and still outside and inside nest [19]. Deceased pups were also collected on a twice-daily basis, up to 21 days (total, 541 pups monitored and 87 found dead), and DNA was collected for genotype analysis. These mice appeared to have died of natural causes. There were no signs that any of these pups were in any distress. The ethics committee at our institution was made aware of the observed unanticipated mortality. Milk spots were present, suggesting that they were feeding adequately. We attempted to determine the cause of lethality in these mice. To assure that this was not related to the mother, foster mothers were utilized, but this did not affect survivability. Autopsy studies did not reveal anatomic changes that could define a precise cause of death.

Individual litters were selected for reproductive analysis. Blood samples from select male mice were taken for testosterone analysis, as described below. Each mouse was sacrificed by means of carbon dioxide inhalation, weighed, and dissected, major tissue types were harvested, and major organs were weighed and then snap-frozen for future assays. Brain, skeletal muscle, heart, lungs, femur, small bowel, colon, liver, spleen, and epidermis were harvested and weighed, prepared for tests as described above, and stained with hematoxylin and eosin. Slides were reviewed by a pathologist blinded to the genotype of each specimen. Male reproductive tract organs were harvested and weighed from wild-type, heterozygote, and mutant littermates at 2, 3, 4, 5, 6, and >8 weeks.

### Testosterone assay

Blood was drawn via direct cardiac puncture from adult *DCUN1D1*^+/+^, *DCUN1D1*^+/-^, and *DCUN1D1*^-/-^ mice (<6 months old) during the evening, when mice are most sexually active. Isolated serum was then assayed for testosterone levels by use of a specific enzyme immunoassay kit (Diagnostic Systems Laboratories).

### Histologic, immunohistochemical, and immunofluorescence analysis

Testes, epididymides, and other organs were dissected, washed with phosphate-buffered saline (PBS), and fixed in either Bouin’s solution or 4% paraformaldehyde at 4°C overnight. Paraformaldehyde-fixed tissues were washed with PBS for 20 min, 3 times, and placed in 70% ethyl alcohol (EtOH) at 4°C for storage. Bouin’s-fixed specimens were washed in 70% EtOH for several days at 4°C, until the wash cleared, and then were stored at 4°C. Tissues were dehydrated with an EtOH gradient, treated with histoclear and paraffin, and embedded in paraffin molds. Five-micrometer sections were prepared, the wax was removed, and the specimens were stained with periodic acid–Schiff or hematoxylin and eosin. Sections were viewed using conventional light microscopy.

For bromodeoxyuridine (BrdU) staining, mice were injected intraperitoneally with 100 mg of BrdU (Sigma) per kg body weight. After 2 h, we dissected and fixed the testes in 4% paraformaldehyde and processed the tissue in paraffin, as described above.

For immunohistochemical analysis, normal mouse immunoglobulin G (IgG) and normal rabbit IgG were used as negative controls. Sections for staining with 4′,6-diamidino-2-phenylindole (DAPI; Sigma), anti-p27 (BD Transduction Labs), anti-phosphorylated histone H3 (Upstate), anti-Ki-67 (Novacastra), BrdU (Roche), anti-Ub (P4D1; Santa Cruz), and anti-LC3 (Cell Signaling Technology) were fixed and processed as described elsewhere [20]. For immunofluorescence analysis, isolated sperm were stained with anti-β-tubulin (Sigma), anti-pericentrin (Abcam), and MitoTracker Red (Invitrogen). Whole slides were scanned using the Mirax Scanner (Carl Zeiss) with a 20x 0.8 numerical aperture objective. Confocal imaging was performed using a Leica TCS SP2 system with a 20x 0.7 numerical aperture water-immersion objective and a 63x 1.2 numerical aperture water-immersion objective.

### In vivo and in vitro neddylation assay

To prepare tissue lysate, mouse tissue was frozen in liquid nitrogen and then crushed into a fine powder using a mortar and pestle. For every 100 μL of tissue powder, 400 μL of tissue lysis buffer (20 mM Tris-HCl, pH 8.0, 137 mM NaCl, 10% glycerol, 1% IGEPAL, 0.1% sodium dodecyl sulfate [SDS], 0.5% NaDeoxycholate, 4 mM ethylenediaminetetraacetic acid, 1 mM NaF, 1 mM Na_3_VO_4_, and 1 mM Na_2_MoO_4_) was added. Samples were sonicated to break the tissue up further and to shear DNA (2–5 min at a power of ~180 watts in rounds of 10 sec sonication/10 sec rest for each cycle). Samples were kept on ice during sonication. Samples were incubated on ice for 30 min and vortexed every 5 min, before being centrifuged at 10,000x g for 20 min at 4°C, to pellet cell debris. The top fat layer was scooped out, and the supernatant was then transferred to a fresh microfuge tube without disturbing the pellet. For *in vivo* neddylation, tissue lysates were prepared with tissue lysis buffer including 10 μM of ML4924 (an inhibitor of the Nedd8-activating enzyme) and 2 mM of 1,10-orthophenathroline (zinc chelator and COPS5 inhibitor) to inhibit postlysis neddylation and deneddylation. Tissue lysates were directly subjected to immunoblotting for cullin(s). *In vitro* neddylation was performed essentially as described elsewhere [11]. The source of Cullin-ROC1 substrate for *in vitro* neddylation reactions was testes lysate; 50 μg of lysates were added to reactions containing 10 nM of APPBP1/Ubc3, 4 nM of Ubc12, 2 μM of Nedd8, and 4 mM of adenosine triphosphate in neddylation buffer (50 mM Tris-HCl, pH 7.6, 55 mM NaCl, 5 mM MgCl_2_, and 1 mM dithiothreitol). Reactions were incubated at 30°C and stopped with the addition of 6x Laemmli buffer. Proteins were resolved on an SDS–polyacrylamide gel electrophoresis gel and subjected to Western blot analysis.

### Germ cell isolation and purification

Male germ cells were freshly purified from dispersed mouse testes by centrifugal elutriation, as previously described [21]. Following elutriation, there were no contaminating somatic testicular cells in the germ cell populations. Germ cell purity was based on specific cell-type scoring assessments (see percentage in parenthesis). Spermatogonia (70–75), midlate pachytene spermatocytes (92), round spermatids (95), elongating spermatids (80–85), and late spermatid (69)–enriched fractions were separated and collected. Protein was extracted from these cell populations and subjected to Western blot analysis.

### Meiotic-stage distribution in *DCUN1D1*^*-/-*^ and control spermatocytes

Spermatocytes were prepared and analyzed as described elsewhere [22]. In brief, testes were isolated, and tunica was removed. Tubules were then disaggregated and chopped with a razorblade in Dulbecco’s Modified Eagle Medium. Aliquots of the cell solution were transferred to multiwell slides containing hypotonic buffer (0.5% NaCl, pH 8.0) and incubated for 15 min to allow attachment of cells to the slide surface. Cells were fixed in 2% paraformaldehyde (PFA) with 0.03% SDS for 3 min, 2% PFA for an additional 3 min, and then rinsed and allowed to air dry for 10 min. Slides were incubated in 1x block (10x stock: 10% goat serum (Sigma), 3% bovine serum albumin, 0.05% Triton X-100, in PBS), then stained overnight at 4°C with primary antibodies (SCP1 [Novus], SCP3 [Novus], and Ƴ-H2AX [Upstate]) diluted in 10x block. Slides were rinsed and incubated for 2 h at room temperature with secondary antibodies (AlexaFluor-488 and -594 goat anti-mouse and goat anti-rabbit IgG [Molecular Probes]) diluted in 10x block. Slides were rinsed, soaked briefly in 100 ng/mL of DAPI (Sigma), rinsed, and allowed to dry in the dark. Coverslips were mounted with antifade media (1 mg/mL p-phenylenediamine, 1x PBS, 80% glycerol) and sealed with nail polish.

### Sperm isolation and analysis

Cauda epididymides were dissected free from the testes and minced in 1 mL of noncapacitating modification of Whiten’s medium (22 mM 4-[2-hydroxyethyl]-1-piperazineethanesulfonic acid, 1.2 mM MgCl_2_, 100 mM NaCl, 4.7 mM KCl, 1.0 mM pyruvic acid, 5.5 mM glucose, 4.8 mM lactic acid hemicalcium salt) and incubated for 5 min at 37°C to allow for sperm dispersal. Sperm counts and motility were calculated by analyzing 10 μL of sample in a hemocytometer, as described elsewhere [23]. The remaining sample was centrifuged at 800 g for 5 min and washed in PBS, and the pellet was appropriately fixed. Alternatively, sperm was centrifuged through a 40% to 90% Percoll gradient at 15,000 g. Each layer was then washed and fixed in the appropriate buffer for both light microscopy and electron microscopy. For video analysis, sperm was collected as described and stored in noncapacitating modification of Whiten’s medium at 37°C. Samples were imaged with a Zeiss Metamorph (Aviovert 200M Inverted Microscope Stand) widefield inverted microscope and filmed using the Roper Scientific Photocascade 512B camera.

### Transmission electron microscopy

Mice were anesthetized with intraperitoneal injection of Avertin. A small hole was made in the right atria and a 10-cc syringe with a 21 gauge needle was used to flush the circulatory system free of blood with PBS. Using the same syringe, cold electron microscopy (EM) fixative (0.1 M sodium cacodylate-buffered 2.5% glutaraldehyde, 2% formaldehyde, and 0.1% picric acid) was slowly infused through the left ventricle until the tail and hind limbs became rigid. The testes and cauda epididymides were dissected and placed in 10 cc of EM fixative and left overnight at 4°C. Samples were then postfixed in 1% OsO_4_ for 1 h. After a routine ethanol graded dehydration, the samples were cut into small pieces and embedded in Epon812. Ultrathin sections were cut with diamond knives and stained with uranyl acetate and lead citrate. Sperm were harvested as described above. After washing and centrifugation, the sample was covered with 1 cc of cold EM fixative and left overnight at 4°C. The same process as above was followed for embedding and viewing. Images were obtained using a JEM 1200 EX Electron Microscope (JEOL, Tokyo, Japan); 1-μm semithin sections were stained with toluidine blue for light microscopy.

### Cold-field emission scanning electron microscopy

Isolated sperm were fixed in 2.5% glutaraldehyde in 0.1 M cacodylate buffer (pH 9) overnight at 4°C. A droplet of each sperm suspension was then placed on 12-mm round coverslips coated with 0.01% poly-l-lysine and allowed to adhere for 30 min at 4°C. Coverslips were rinsed by dipping them in 0.1 M cacodylate buffer and were dehydrated using an ethanol series (20%, 50%, 70%, 80%, 95%, 2 min each; 100%, 2 changes for 10 min each). Specimens were critical-point dried (Oerlikon Balzers), briefly sputter-coated (10 to 30 sec; Desk II, Denton Vacuum) with gold/palladium, and viewed in a Hitachi S-4700 cold-field emission scanning electron microscope operating at 5 kV accelerating voltage and 7 μA emission current. Images were collected digitally at a resolution of 2560 × 1920 pixels (512 pixels/inch) [24].

## Results

### DCUN1D1^-/-^ mice are viable

**Fig 1:**
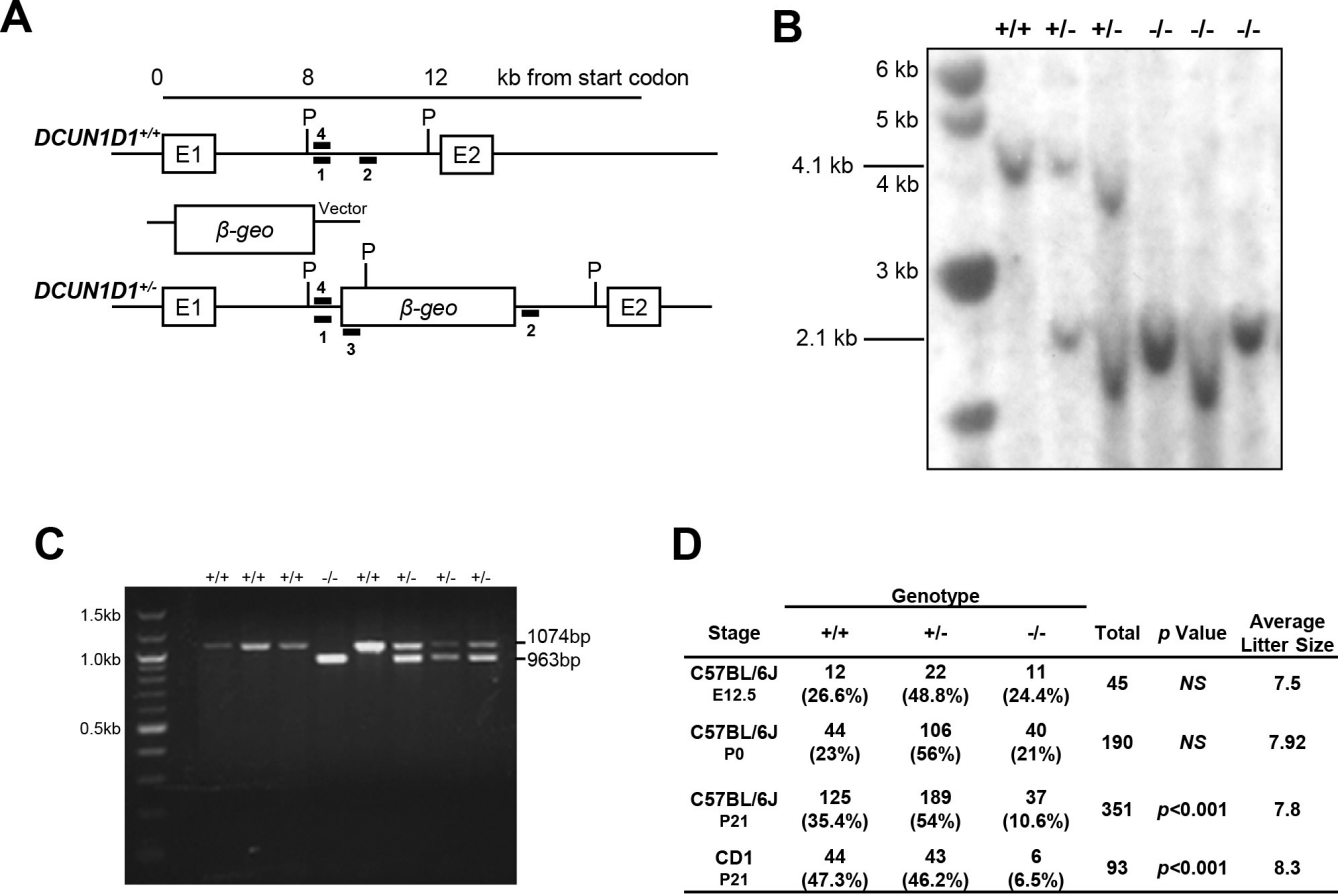
*DCUN1D1*^*-/-*^ mice are viable. (A) Schematic diagram of the mouse *DCUN1D1* gene locus (top), targeting vector (middle, pGT0lxf), and the disrupted mutant allele following homologous recombination (bottom). The targeting vector contains the β-galactosidase coding sequence inserted between exon 1 (E1) and exon 2 (E2) of *DCUN1D1*. The locations of PCR genotyping 5’ forward (1), 3’ reverse (2), and vector-specific reverse (3) primers used to distinguish mutant from wild-type alleles are shown. The location of the 5’ probe (4) used for Southern blotting analysis within the PvuII digestion sites (P) is indicated. (B) Results from Southern blotting of tail DNAs from F1 progeny of heterozygote intercrosses digested with PvuII. A 2.1-kb fragment indicates heterozygote. (C) PCR analysis of DNA extracted from *DCUN1D1*^*+/+*^, *DCUN1D1*^*+/-*^, and *DCUN1D1*^*-/-*^ mice with primers 1, 2, and 3 denoted in Fig 1A. A product of 963 bp indicates heterozygote. (D) Results from mating *DCUN1D1*^*+/-*^ mice, showing normal litter sizes and maintenance of expected Mendelian ratios at E12.5 and birth (P0). There was a reduction in viability in *DCUN1D1*^*-/-*^ mice on postnatal day 21 (P21) in both C57BL/6J and CD1 outbred strains (rows 3 and 4, respectively). *P* values are derived from χ^2^ analyses.

To determine whether neddylation is essential for development in higher organisms, we generated mice with targeted inactivation of *DCUN1D1* using viral-based gene trap mutagenesis (Fig 1A). Germline transmission to F1 heterozygotes was confirmed by Southern blotting analysis of PvuII-digested genomic DNA, which showed the presence of the diagnostic 2.1-kb fragment (Fig 1B). A PCR method was established and validated to facilitate rapid genotyping of the pups (Fig 1C).

Time-dam experiments showed that *DCUN1D1*^*-/-*^ mice developed normally *in utero* and were born in Mendelian ratios, which contrasts the lethality that results from inactivation of *DCN1* in yeast and *Caenorhabditis elegans* [8]. Viability was reduced postnatally, with *DCUN1D1*^*-/-*^ mice composing only 10.6% of pups by 3 weeks (hazard ratio, 1.72 [confidence interval, 1.4–2.0]; *p*<0.001; n = 45 litters) (Fig 1D). Postnatal death of *DCUN1D1*^*-/-*^ mice appeared to result from their inability to compete with littermates. However, use of surrogate mothers and back-crossing into a less aggressive mouse species (CD1) did not alter postnatal mortality, suggesting that physiological factors may contribute to lethality in *DCUN1D1*^*-/-*^ mice (Fig 1D). Gross and histopathologic examination of brain, heart, lung, spleen, liver, kidney, skeletal muscle, bone marrow, skin, testis, and ovary revealed no differences between *DCUN1D1*^*+/+*^, *DCUN1D1*^*+/-*^, and *DCUN1D1*^*-/-*^ newborn mice that could explain the reduced viability and runting among *DCUN1D1*^*-/-*^ mice.

The only phenotypic difference among the mice was runting in *DCUN1D1*^*-/-*^ mice [10]. The smaller size of *DCUN1D1*^*-/-*^ mice was independent of sex or genetic background, as both males and females were smaller and mice produced in the CD1-outbred background were also runted. We have previously shown that proliferative activity is decreased in murine embryonic fibroblasts from *DCUN1D1*^*-/-*^ mice, owing to defective cullin neddylation—this may also be the cause of runting in these mice [10].

### DCUN1D1^-/-^ mice are infertile due to defective spermatogenesis

**Fig 2:**
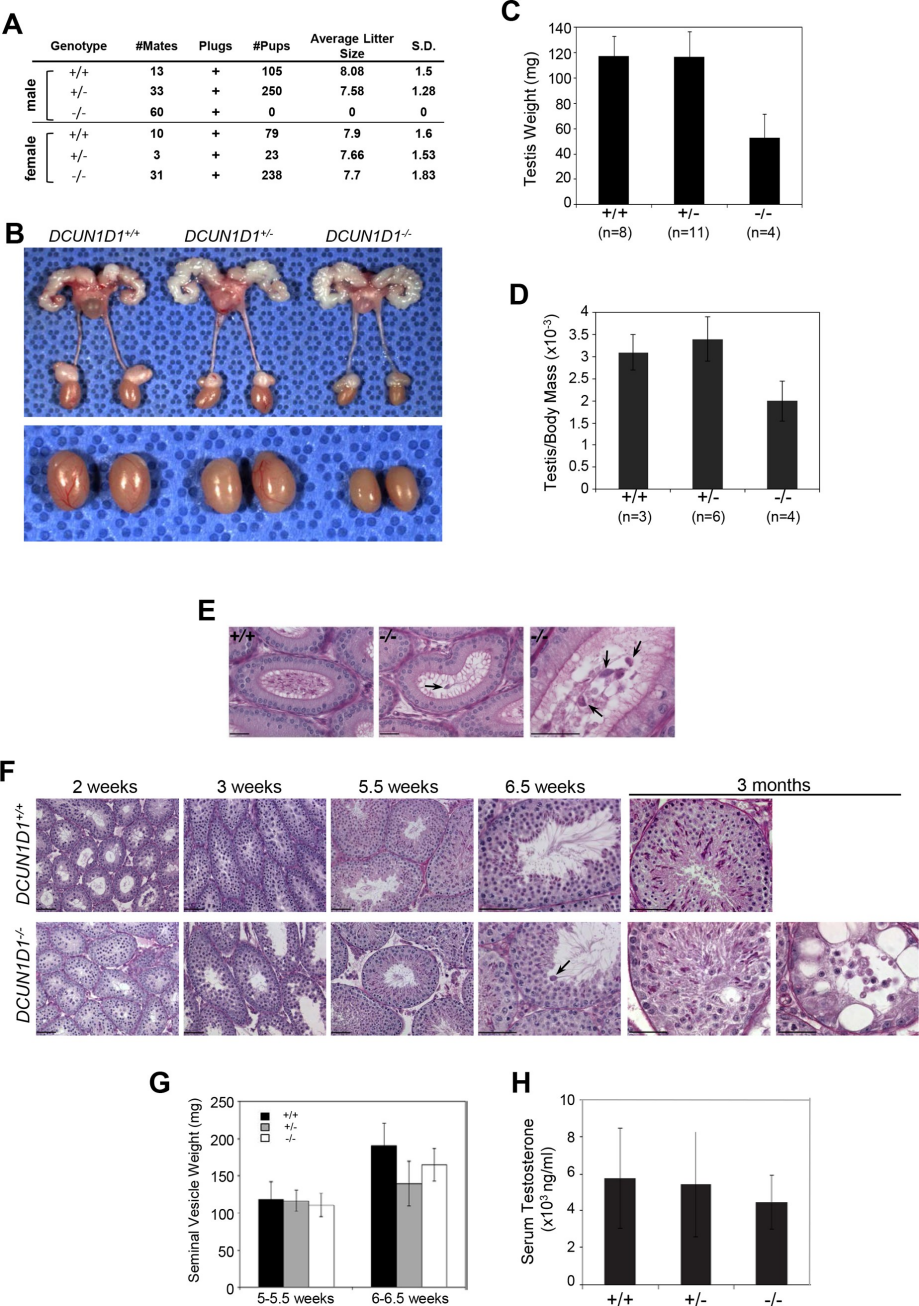
***DCUN1D1***^***-/-***^ **mice are infertile due to defective spermatogenesis.** (A) Table showing results from mating mice of different *DCUN1D1* genetic backgrounds (Genotypes) with fertile wild-type C57BL/6J mice. The data show the number of matings assessed (#Mates), confirmation of copulation by presence of vaginal plugs in females (Plugs), total number of pups derived (#Pups), and average litter size per mating, with standard deviation (S.D.). Matings with *DCUN1D1*^*-/-*^ males did not result in any viable pregnancies or offspring (row 3). (B-D) Reproductive tract from 3-month-old *DCUN1D1*^*+/+*^, *DCUN1D1*^*+/-*^, and *DCUN1D1*^*-/-*^ male littermates, showing reduced testicular size in *DCUN1D1*^*-/-*^ mice but no differences in size of seminal vesicles or other anatomical defects in the reproductive tract. The lower panel of Fig 2B shows a close-up view of testis. (E) Periodic acid–Schiff (PAS)–stained sections of caudal epididymis from *DCUN1D1*^*+/+*^ and *DCUN1D1*^*-/-*^ mice at 6.5 weeks, showing a paucity of mature spermatozoa and the presence of large, macrocephalic spermatozoa (arrow) in the cauda epididymis of *DCUN1D1*^*-/-*^ mice (scale bar, 25 μm). (F) PAS-stained cross-sections of seminiferous tubules from littermate *DCUN1D1*^*+/+*^ (top row) or *DCUN1D1*^*-/-*^ mice (bottom row) at different ages, showing asynchronous spermatogenesis with multiple generations of developing spermatids appearing at 5.5 weeks, abnormal sperm in the seminiferous lumen by 6.5 weeks (arrow), and progressive degeneration with desynchronization (column 5, bottom) and vacuolization with loss of germ cells (column 6, bottom) in 3-month-old *DCUN1D1*^*-/-*^ mice (scale bar, 50 μm). (G) Weights of seminal vesicles from *DCUN1D1*^*+/+*^, *DCUN1D1*^*+/-*^, and *DCUN1D1*^*-/-*^ mice were measured in milligrams (n = 8). No obvious differences were seen in mice at 5 to 5.5 or 6 to 6.5 weeks of age. As development of seminal vesicles is dependent on testosterone, the lack of an observed difference indicates the presence of normal testosterone levels in *DCUN1D1*^*-/-*^ mice. (H) Normal testosterone in *DCUN1D1*^*-/-*^ mice. Blood was extracted via direct cardiac puncture from adult *DCUN1D1*^+/+^, *DCUN1D1*^+/-^, and *DCUN1D1*^-/-^ mice during the evening (n = 8), when mice are most sexually active. Testosterone levels were measured as described in Experimental Procedures. Mean (±SD) serum testosterone levels were similar among *DCUN1D1*^*-/-*^, *DCUN1D1*^*+/+*^, and *DCUN1D1*^*+/-*^ males.

In contrast to yeast and *C*. *elegans*, in which deletion of *DCN1* results in lethality, the viability of *DCUN1D1*-knockout mice suggests that *DCUN1D1*’s function either is nonessential or is compensated for by paralogs that are exclusively present in higher organisms [8, 10]. To address this issue, we assessed testicular development in *DCUN1D1*^*-/-*^ mice, in which expression of *DCUN1D1* is disproportionately higher than that of *DCUN1D2 (SCCRO2)*, its most closely related paralog [17]. Mating of *DCUN1D1*^*-/-*^ males with wild-type females did not yield any pregnancies, despite normal copulatory behavior (confirmed by the presence of vaginal plugs)—this suggests *DCUN1D1*^*-/-*^ males are infertile (Fig 2A). In contrast, *DCUN1D1*^*-/-*^ females and *DCUN1D1*^*+/-*^ males produced normal-size litters, establishing their fertility (Fig 2A). Testes from adult *DCUN1D1*^*-/-*^ mice were markedly atrophic, compared with those from *DCUN1D1*^*+/+*^ and *DCUN1D1*^*+/-*^ mice, even after controlling for differences in body weight (Fig 2B–2D). The reduced numbers of sperm within the epididymis, combined with their limited motility and severe structural defects, suggest the presence of a defect in spermatogenesis in *DCUN1D1*^*-/-*^ mice (Fig 2E and S1 and S2 Movies). Consistent with these observations, histologic analysis of testes from 3-month-old *DCUN1D1*^*-/-*^ mice revealed marked abnormalities and asynchronous spermatogenesis, with multiple generations of developing spermatids in the same cross-section of the seminiferous tubule (Fig 2F). Degeneration of the seminiferous tubule epithelium was also evident, ranging from sloughing into the lumen of the tubule to development of large vacuoles and a Sertoli cell–only phenotype with complete loss of germ cells (Fig 2F). However, the timing and histologic pattern of changes suggest that the progressive depletion of spermatids and their precursors is likely secondary to disrupted inter- and intracellular communication resulting from failed spermatogenesis [25]. The distal reproductive tract, including the prostate and seminal vesicles, was normal in *DCUN1D1*^*-/-*^ males (Fig 2G, n = 8). In addition, testosterone levels were normal in *DCUN1D1*^*-/-*^ mice, suggesting that infertility was not caused by hormonal deficiencies (Fig 2H, n = 8).

### The first wave of mitosis and meiosis is normal in testes from *DCUN1D1*^*-/-*^ mice

**Fig 3:**
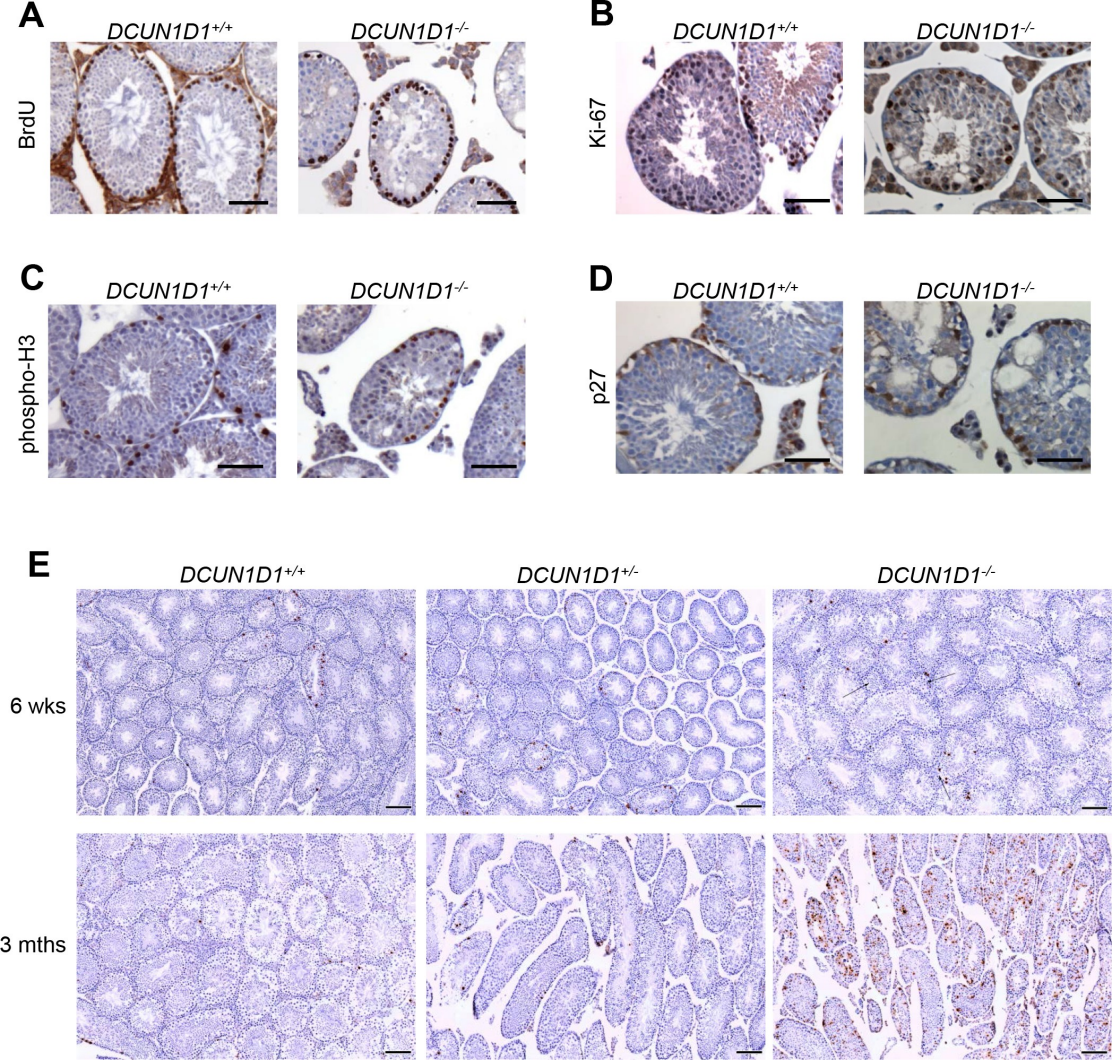
First wave of mitosis is normal in testes from *DCUN1D1*^*-/-*^ mice. (A, B, and C) Spermatogonia from *DCUN1D1*^*-/-*^ mice have normal proliferative activity. Representative results from immunostaining of testis sections, showing similar patterns of BrdU (A), Ki-67 (B), and phospho-histone H3 (C) expression in spermatogonia from *DCUN1D1*^*-/-*^ and *DCUN1D1*^+/+^ mice, which suggests that mitotic activity is intact (scale bar, 50 μm). (D) Sertoli cell numbers are normal in *DCUN1D1*^*-/-*^ mice. Immunostaining for p27, which is exclusively expressed in Sertoli cells, confirmed that Sertoli cells were present in adequate number in *DCUN1D1*^*-/-*^ mice (23.5±8.0 per section, n = 8), compared with *DCUN1D1*^*+/+*^ mice (25.5±8.5 per section, n = 6) (scale bar, 50 μm). (E) Results of TUNEL assays on testis sections from *DCUN1D1*^*+/+*^ (left panels), *DCUN1D1*^*+/-*^ (middle panels), and *DCUN1D1*^*-/-*^ (right panels) mice at 6 weeks (top panels) and 3 months (bottom panels) of age. Apoptosis was increased only in testis from 3-month-old *DCUN1D1*^*-/-*^ mice (scale bar, 100 μm).

**Fig 4:**
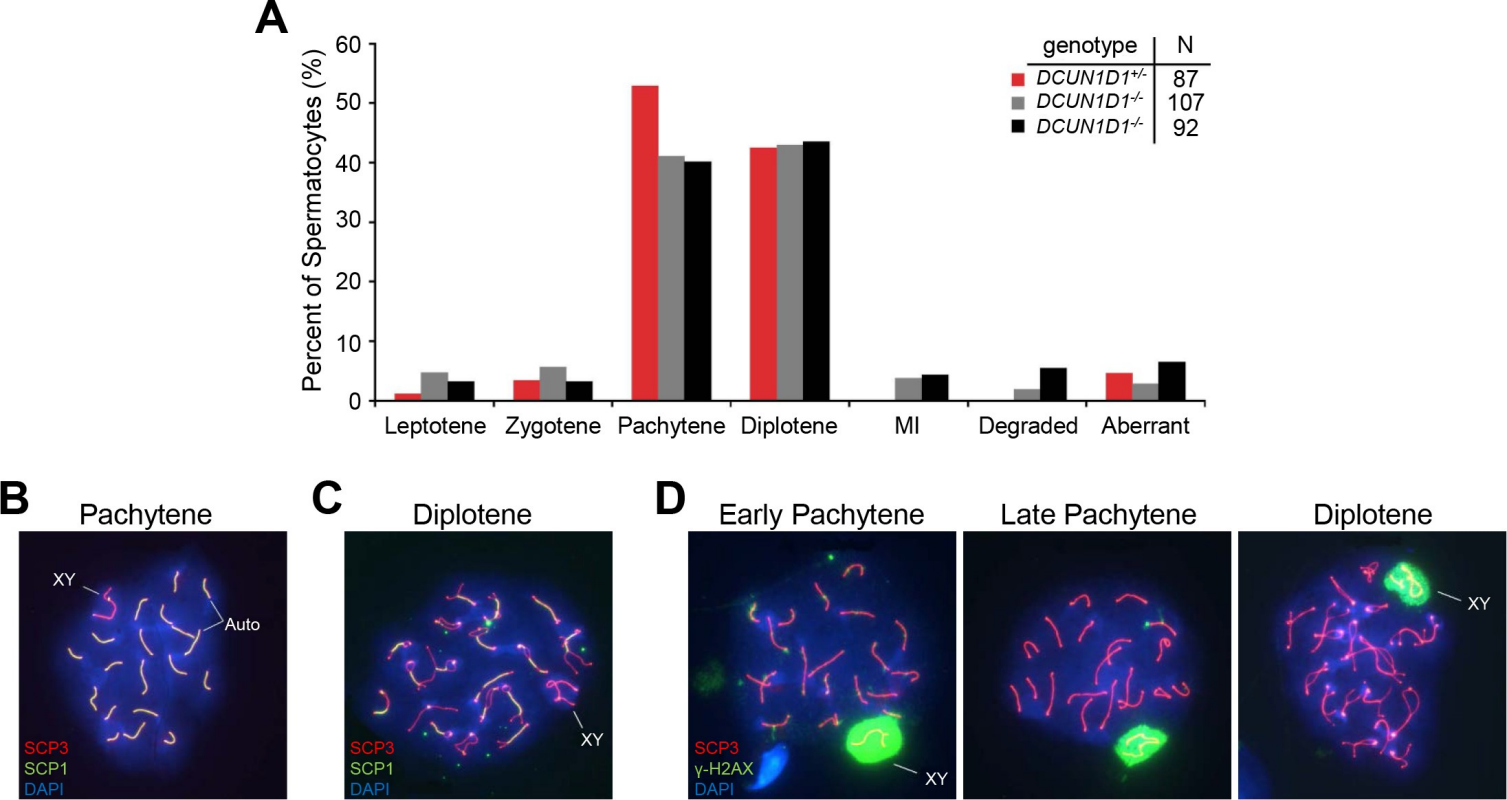
Meiosis is not aberrant during spermatogenesis in *DCUN1D1*^*-/-*^ mice. (A) Meiotic stage distribution in mutant and control spermatocytes. Spermatocyte spreads from 6-week-old mice were stained with SCP3 and SCP1 to analyze synaptonemal complex (SC) morphogenesis and were categorized according to meiotic stage. No obvious defects in meiosis were found in testes from *DCUN1D1*^*-/-*^ mice, compared with *DCUN1D1*^+/+^ mice. MI, metaphase I. Normal pachytene spermatocytes (B), diplotene spermatocytes (C), and XY body (D) from *DCUN1D1*^*-/-*^ mice identified by SCP3, SCP1, and γ-H2AX staining. Auto, autosomes.

The first wave of spermatogenesis in mice occurs in a synchronized, chronologically predictable manner, starting with mitosis in spermatogonia and meiosis in primary spermatocytes followed by differentiation of round and elongating spermatids to produce mature spermatozoa. Accordingly, defining the time of the onset of developmental defects during spermatogenesis would help to identify the processes and associated molecular pathways involved. Gross evaluation showed that testicular size and weight were normal until 6 weeks of age in *DCUN1D1*^*-/-*^ mice. Histologic analysis of testicular sections from *DCUN1D1*^*-/-*^ mice showed that seminiferous tubules developed normally until 5 weeks of age (Fig 2D). This suggests that pituitary-gonadal access is intact, as the first wave of spermatogenesis is initiated at the appropriate age. Moreover, the absence of any detectable histologic defects until 5 weeks of age suggests that mitosis and meiosis progress normally in *DCUN1D1*^*-/-*^ mice. Immunostaining of spermatogonia showed normal patterns of BrdU, Ki-67, and phospho-histone H3 expression, suggesting that mitotic activity is intact in *DCUN1D1*^*-/-*^ mice (Figs 3A–3C). Moreover, immunostaining for p27 confirmed that Sertoli cells were present in adequate number (Fig 3D). The lack of apoptosis detected by TUNEL staining (Fig 3E) and conservation of germ cell numbers confirmed that germ cells are normally maintained in testes from juvenile *DCUN1D1*^*-/-*^ mice. In contrast, an increase in apoptosis was seen in germ cells in adult *DCUN1D1*^*-/-*^ mice. This likely developed in response to defective progression of spermatogenesis, rather than as a primary defect in germ cells. Previous studies have shown that inactivation of the *DCUN1D1* ortholog *DCN1* in yeast is associated with a decrease in Cul3-mediated ubiquitination and subsequent turnover of MEI1 (Katanin p60 in mouse), leading to meiotic dysfunction [8]. In order to specifically confirm the absence of a meiotic defect, we examined meiotic spreads in testes from *DCUN1D1*^*-/-*^ mice; we found no abnormalities relative to *DCUN1D1*^*+/-*^ mice (Fig 4A–4D). These observations map the onset of abnormalities to postmeiotic spermatogenesis, suggesting that a defect in terminal differentiation is present in *DCUN1D1*^*-/-*^ mice.

### Loss of DCUN1D1 causes defects in spermiogenesis

**Fig 5:**
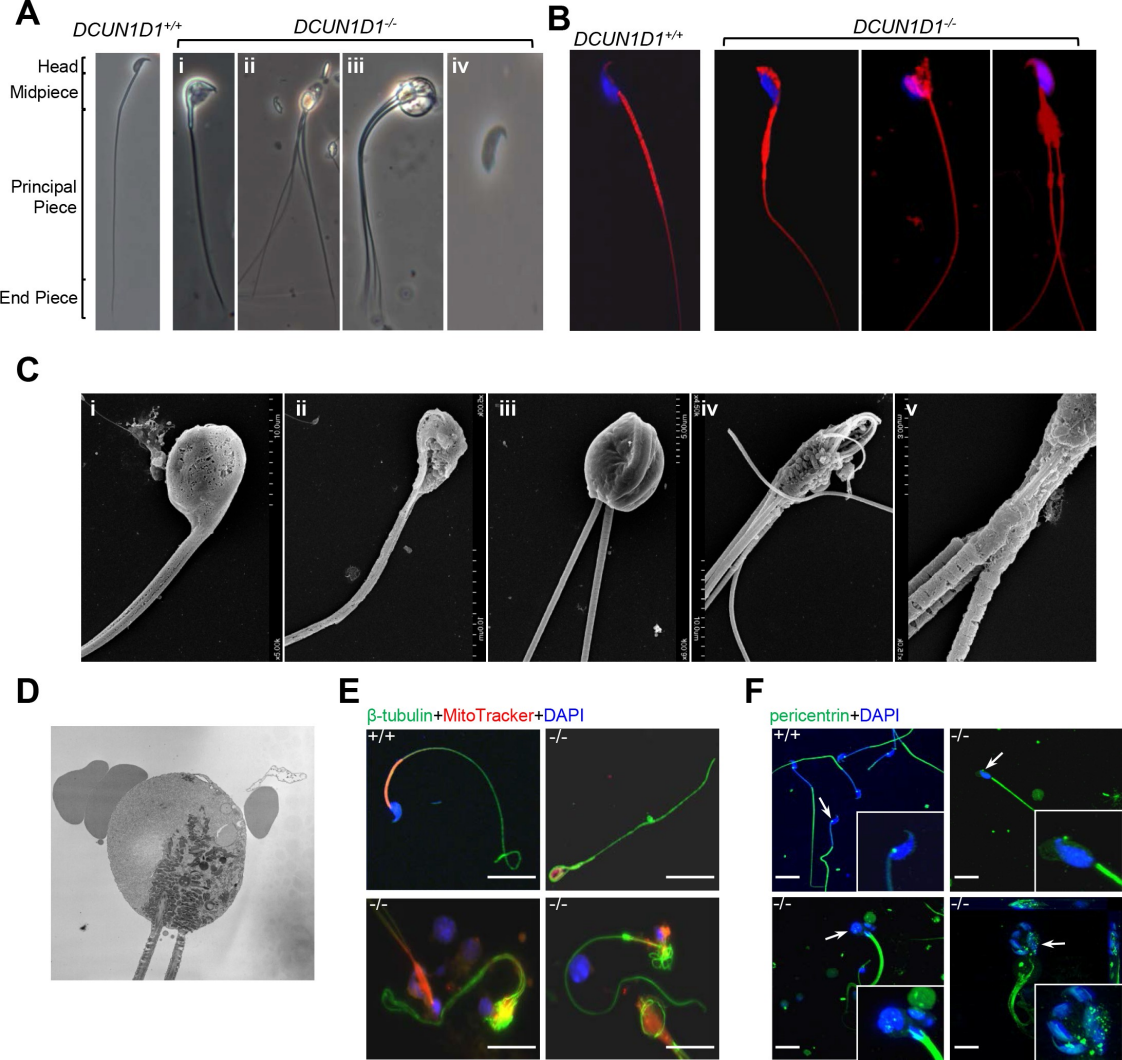
Loss of DCUN1D1 causes defects in spermiogenesis. (A) Bright-field images of sperm from *DCUN1D1*^*+/+*^ and *DCUN1D1*^*-/-*^ mice, as indicated. The range of abnormalities in sperm from *DCUN1D1*^*-/-*^ mice includes macrocephalic head (i, ii, and iii), abnormal midpiece (i, ii, and iii), multiple flagella (ii and iii), retention of cytoplasm with accumulation of vacuoles (iii), and detached head (iv). (B) Results from MitoTracker Red staining, showing the presence of normal mitochondrial activity but abnormal mitochondrial position in *DCUN1D1*^*-/-*^ sperm. (C) Representative scanning electron micrographs of spermatozoa from *DCUN1D1*^*-/-*^ mice, showing abnormal morphologic appearance: globozoospermia or macrocephaly (i, ii, and iii), multiple flagella (iii, iv, and v), abnormal midpiece (iii, iv, and v), abnormal mitochondrial sheath with malpositioned mitochondria (iv-v), and exposed axoneme (v). (D) Transmission electron micrographs showing spermatozoa with multiple flagella, abnormal mitochondrial position and excessive cytoplasm. (E) Immunostaining of caudal spermatozoa for β-tubulin, showing a normal expression pattern in sperm from *DCUN1D1*^*+/+*^ mice and redundant loops of microtubules within the macrocephalic heads of sperm from *DCUN1D1*^*-/-*^ mice (scale bar, 10 μm). (F) Immunostaining for pericentrin, showing normal centrioles in spermatozoa from *DCUN1D1*^*+/+*^ mice but malpositioned and supernumerary centrioles (white arrow) in spermatozoa from *DCUN1D1*^*-/-*^ mice (scale bar, 10 μm).

**Fig 6:**
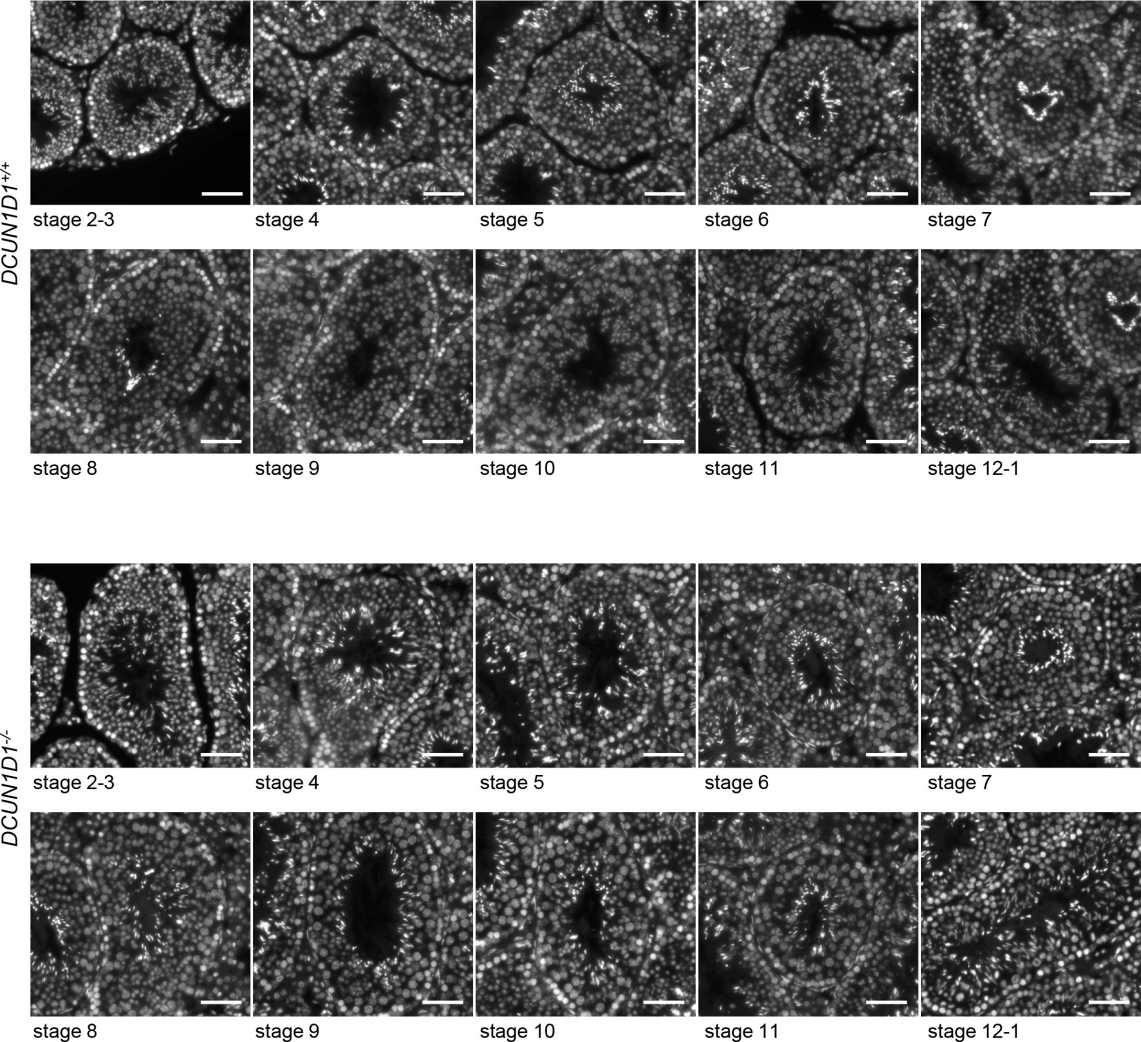
Defect of spermiation in *DCUN1D1*^*-/-*^ mice. Testis sections from *DCUN1D1*^*+/+*^ and *DCUN1D1*^*-/-*^ mice at 6 weeks old were stained with DAPI and staged. An increase in the intensity of the DAPI signal is consistent with progressive condensation of nuclei during spermatid development (see the spermatids in the luminal aspect of the tubules). Testes from *DCUN1D1*^*+/+*^ mice show normal release of mature spermatozoa into the lumen of seminiferous tubules starting from stage 8. In contrast, the majority of spermatozoa with condensed nuclei in *DCUN1D1*^*-/-*^ mice are not released, resulting in the presence of two generations of developing spermatids coexisting in a single tubule (stage 9 to 12).

Abnormal spermatids were first detected in the developing seminiferous tubule, tubular lumen, and epididymis by 5.5 weeks in *DCUN1D1*^*-/-*^ testis, temporally correlating with onset of spermiogenesis (Fig 2D). During spermiogenesis, round spermatids undergo significant morphologic changes to mature into spermatozoa. These include nuclear condensation, development of the tail, organization of the mitochondria at the midpiece, formation of a residual body to discard nonessential cytosolic materials, and resolution of intercellular bridges (individualization in *Drosophila*). Upon completion of spermiogenesis, mature spermatozoa are released into the seminiferous lumen. To begin to identify the defect in spermiogenesis, we assessed the morphologic appearance of spermatozoa in *DCUN1D1*^*-/-*^ mice. Multiple abnormalities were seen in spermatozoa in *DCUN1D1*^*-/-*^ mice (99.5%, n = 241), affecting the sperm head (222), midpiece (73), and tail (157). Globozoospermia (195), macrocephaly (135), and multiple flagella (95) were present in many spermatozoa, suggesting a lack of cytoplasmic condensation and/or proper cell separation (Fig 5A). Furthermore, detached heads (49) were significantly more common in semen samples from *DCUN1D1*^*-/-*^ mice than in *DCUN1D1*^*+/+*^ and *DCUN1D1*^*+/-*^ cauda epididymis, suggesting that the connection between the head and the tail is abnormally fragile (Fig 5A). The mitochondrial sheath was disorganized, with mitochondria scattered in varied locations, although the mitochondria retained normal function, as shown by positive staining on MitoTracker assay (Fig 5B). The structural defects in spermatozoa in *DCUN1D1*^*-/-*^ mice were also evident by scanning electron microscopy (Fig 5C). Transmission electron microscopy confirmed abnormal head, heads composed of several nuclei, multiple flagella within the same membrane, lack of cytoplasmic reduction, and abnormal positioning of mitochondria (Fig 5D). Tubulin staining of mature spermatozoa in *DCUN1D1*^*-/-*^ mice also revealed multiple abnormalities, including the presence of globozoospermia, macrocephaly, and multiple flagella (Fig 5E). The malformed spermatozoa in *DCUN1D1*^*-/-*^ mice were multinucleated, with supernumerary and malpositioned centrioles, as observed by DAPI staining and immunostaining for pericentrin, respectively (Fig 5F). Finally, whereas wild-type mice started to release mature spermatozoa into the seminiferous lumen by stage 8, knockout mice failed to do so—therefore, very few spermatozoa were found in the epididymis (Fig 6). Combined, these findings suggest the presence of multiple defects, including resolution of intercellular bridges, during spermiogenesis in *DCUN1D1*^*-/-*^ mice.

### Cullin neddylation is reduced in testes from *DCUN1D1*^*-/-*^ mice

**Fig 7:**
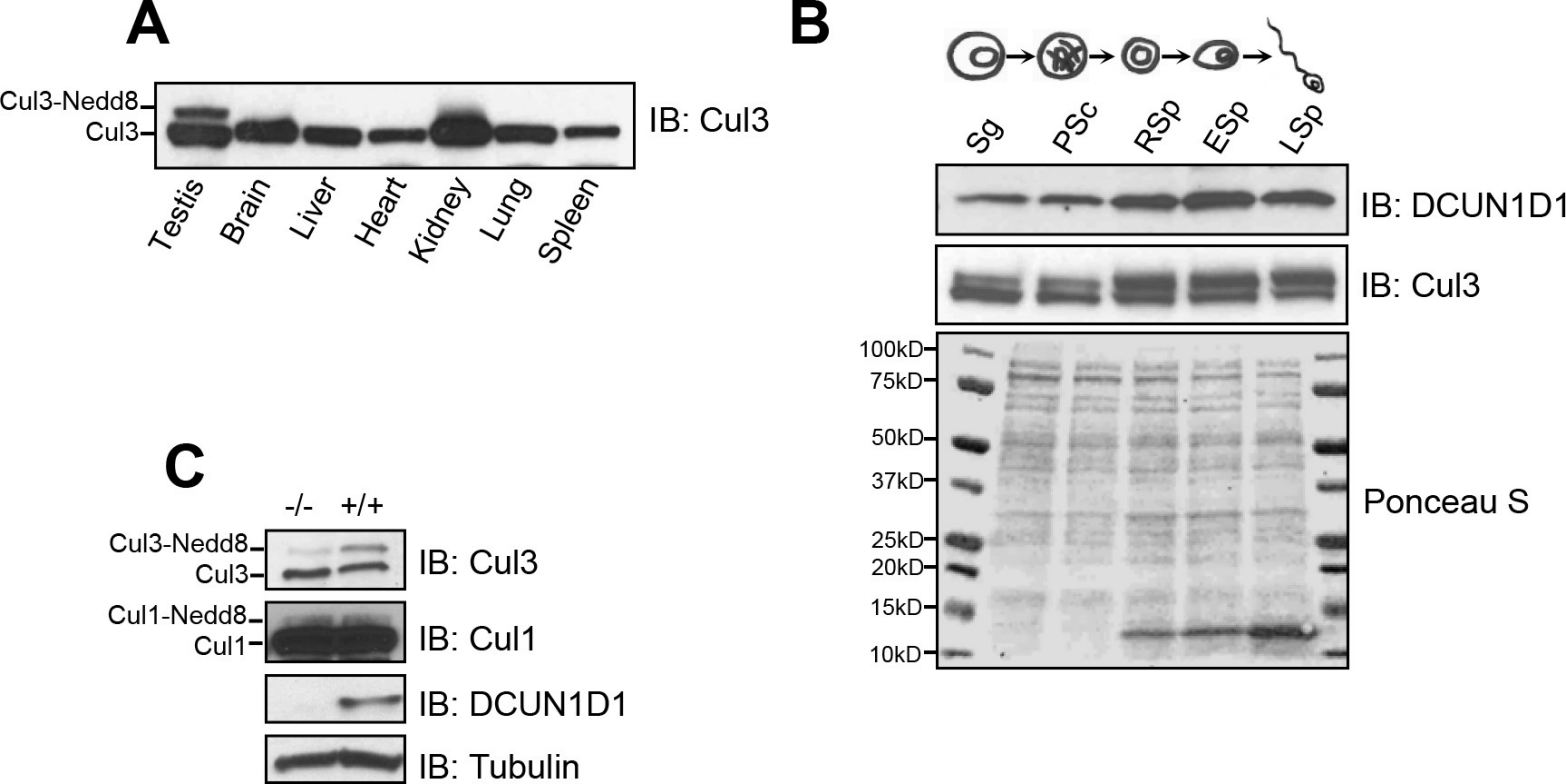
Cullin neddylation is reduced in testes from *DCUN1D1*^*-/-*^ mice. (A) Immunoblot of lysates (50 μg) from different organs from wild-type mice, showing that the highest basal level of neddylated Cul3 is in the testis. (B) Western blotting analysis of elutriated germ cell fractions, showing higher levels of DCUN1D1 (row 1) and neddylated Cul3 (row 2) in round spermatids (RSp), elongating spermatids (ESp), and late spermatids (LSp), compared with spermatogonia (Sg) and pachytene spermatocytes (PSc). Ponceau S staining confirmed equal loading of elutriated fractions. (C) Western blotting analysis of testis lysates, showing a marked reduction in neddylated Cul3 (upper band) levels in testis lysates from *DCUN1D1*^*-/-*^ mice, compared with *DCUN1D1*^*+/+*^ mice. Note that DCUN1D1 is not expressed in lysates from *DCUN1D1*^*-/-*^ mice.

To evaluate whether DCUN1D1 is required for neddylation in the testis, we assessed the levels of neddylated cullins in different organs from wild-type and *DCUN1D1*^*-/-*^ mice. In wild-type mice, the highest steady-state level of neddylated Cul3 was observed in the testis (Fig 7A). To begin to determine whether DCUN1D1 regulates neddylation of Cul3 in the testis, we assessed DCUN1D1 and neddylated Cul3 levels during progressive stages of spermatogenesis. Immunoblotting showed that levels of DCUN1D1 increased during the terminal stages of spermatogenesis in elutriated testis fractions from wild-type mice (Fig 7B). Analysis of the same elutriated fractions showed that levels of neddylated Cul3 temporally correlated with levels of *DCUN1D1* expression, increasing during the terminal stages of spermatogenesis (Fig 7B). Furthermore, immunoblotting showed reduced levels of neddylated Cul3 in testicular lysates from littermate *DCUN1D1*^*-/-*^ mice (but not in lysates from other organs), compared with those from *DCUN1D1*^*+/+*^ mice (Fig 7C). Severe morphologic defects in sperm development in *DCUN1D1*^*-/-*^ mice precluded reliable separation of sperm by elutriation, thus limiting our ability to map the specific time during spermatogenesis when Cul3 neddylation was reduced. As we reported before, defective Cul3 neddylation in testis lysates from *DCUN1D1*^*-/-*^ mice was rescued by the addition of recombinant DCUN1D1, but not DCUN1D1^D241A^, a mutant that loses neddylation activity [17]. Consistent with its high degree of domain and sequence conservation, the addition of DCUN1D2, but not DCUN1D4 or DCUN1D5, rescued Cul3 neddylation in testis lysates from *DCUN1D1*^*-/-*^ mice [17].These findings confirm that loss of *DCUN1D1* results in decreased neddylation of Cul3 in the testis. Furthermore, it appears that DCUN1D1 mediates the neddylation of Cul3 during spermatogenesis and that its functions in other organs, in which *DCUN1D2* is coexpressed with *DCUN1D1*, may be compensated for by DCUN1D2, its most closely related paralog.

### Ubiquitination is reduced in testes from *DCUN1D1*^*-/-*^ mice

**Fig 8:**
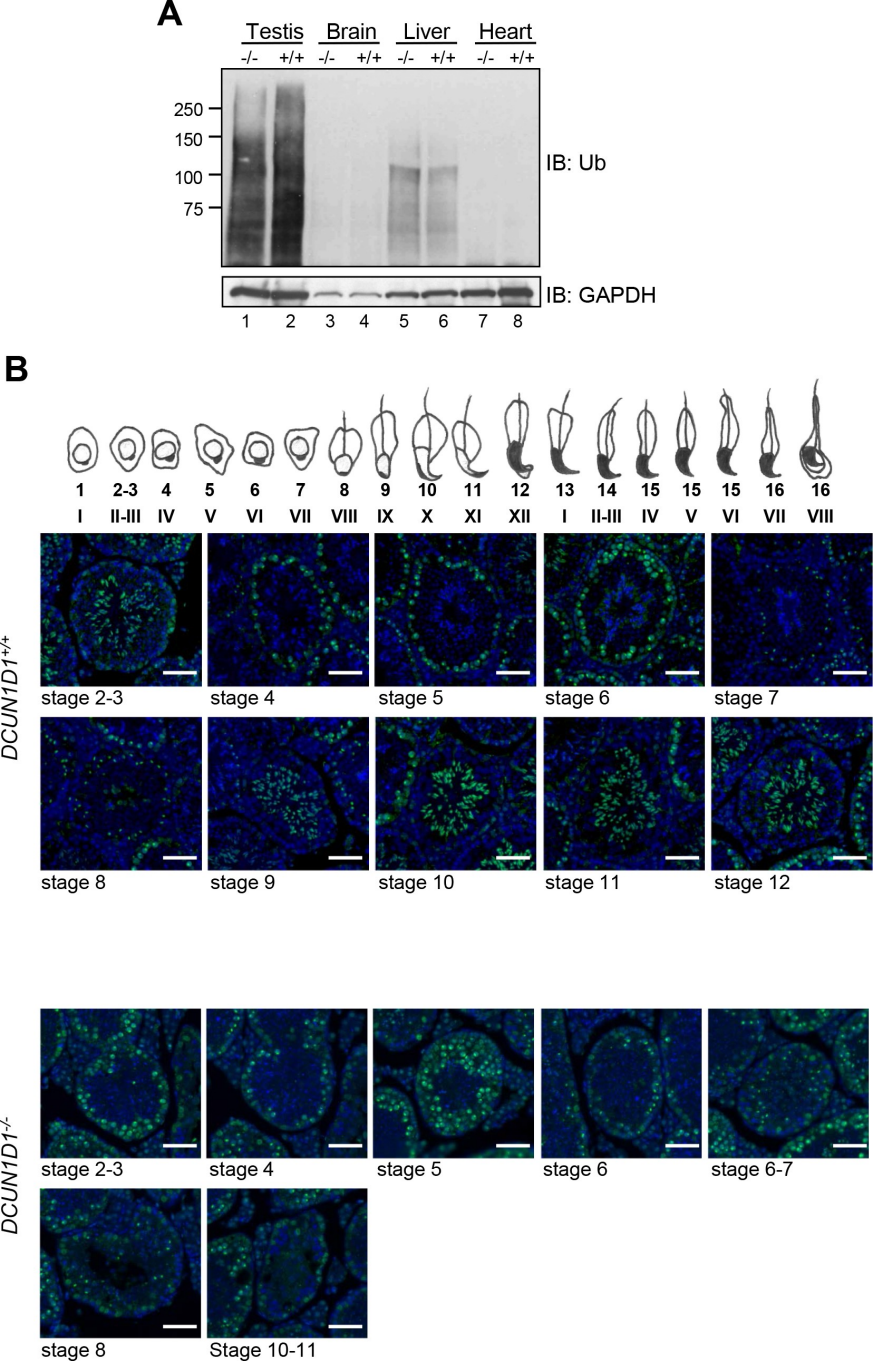
Ubiquitination is reduced in testes from *DCUN1D1*^*-/-*^ mice. (A) Western blotting analysis of lysates of different tissues from *DCUN1D1*^*+/+*^ and *DCUN1D1*^*-/-*^ mice, using antibody against polyubiquitin chains, showing a reduction in ubiquitinated proteins in testes from *DCUN1D1*^*-/-*^ (lane 1) compared with *DCUN1D1*^*+/+*^ (lane 2) mice, but not in other organs. (B) A sketch of staged mouse spermatogenesis is shown at the top. Immunofluorescence using antibody against polyubiquitin chains, showing accumulation of ubiquitinated proteins in testes from *DCUN1D1*^*+/+*^ mice in the sex body and the nucleus of pachytene spermatocytes (see the periphery of tubules) and in the cytoplasm of spermatids at stage 2 to 3 and 9 to 12 (luminal aspect of tubule) (upper two rows). Although ubiquitination in meiotic pachytene spermatocytes is intact, *DCUN1D1*^*-/-*^ mice show no accumulation of ubiquitinated proteins in developing spermatids (scale bar, 50 μm).

It is well-established that DCUN1D1-mediated neddylation promotes assembly and activity of CRL-type ubiquitination E3 complexes. To determine whether knockout of *DCUN1D1* in mice affects ubiquitination, we assessed levels of total ubiquitinated proteins by immunoblotting lysates from different organs from littermate *DCUN1D1*^*-/-*^ and *DCUN1D1*^*+/+*^ mice using an antibody against polyubiquitin chains. The total pool of ubiquitinated proteins was lower in testis lysates from *DCUN1D1*^*-/-*^ mice than in those from *DCUN1D1*^*+/+*^ mice, but not in other organs tested (Fig 8A). To validate the association between ubiquitination and defective neddylation in testes from *DCUN1D1*^*-/-*^ mice, we mapped the patterns of accumulation of ubiquitinated proteins during different stages of sperm development by immunostaining testis sections from littermate *DCUN1D1*^*+/+*^ and *DCUN1D1*^*-/-*^ mice with antibody against polyubiquitin chains. In *DCUN1D1*^*+/+*^ mice, polyubiquitinated proteins accumulated in two discrete patterns during spermatogenesis: in the nucleus of pachytene spermatocytes, with the most intense signal in the sex body, and, subsequently, in the cytoplasm of the elongating spermatids at stage 2 to 3 and stage 9 to 12 (Fig 8B). Whereas accumulation of polyubiquitinated proteins was retained in pachytene spermatocytes in *DCUN1D1*^*-/-*^ mice, there was a near complete loss of accumulation of polyubiquitinated proteins in differentiating spermatids during the same stages when DCUN1D1 expression was the highest in wild-type mice. Taken together, these findings suggest that ubiquitination during the final stages of spermatogenesis is regulated by DCUN1D1-promoted cullin neddylation.

## Discussion

*DCUN1D1*^*-/-*^ mice have proven to be an excellent model to dissect neddylation-related pathways. Using this model, we showed that inactivation of DCUN1D1 results in prolonged mitotic time due to delayed and/or failed abscission in murine embryonic fibroblasts [26]. The effects of DCUN1D1 on abscission involve its role in neddylation and localization of Cul3 to the midbody. The Cul3 adaptor KLHL21 mediates DCUN1D1’s effects on abscission, as it fails to localize to the midbody in DCUN1D1-deficient cells during abscission and its inactivation results in phenotypic changes identical to those observed with DCUN1D1 inactivation. Ubiquitination-promoted turnover of Aurora B at the midbody is deficient in DCUN1D1- and KLHL21-deficient cells, suggesting that it is the target of Cul3^KLHL21^ at the midbody. The correction of abscission delays in DCUN1D1-deficient cells with the addition of Aurora B inhibitor at the midbody stage suggests that Aurora B is the target of DCUN1D1-promoted Cul3^KLHL21^ activity. The activity of other Cul3-anchored complexes, including Cul3^KLHL9/KLHL13^, is intact in DCUN1D1-deficient cells, suggesting that DCUN1D1 selectively, rather than collectively, neddylates cullins *in vivo*. Combined, these characteristics support a model in which DCUN1D1, the substrate, and substrate adaptors cooperatively provide tight control of neddylation and CRL activity *in vivo*.

Observed defects in sperm development in *DCUN1D1*^*-/-*^ mice provided an opportunity to determine the role of neddylation *in vivo*. The range of phenotypic changes seen in spermatozoa from *DCUN1D1*^*-/-*^ mice suggests the presence of a defect in a process essential for differentiation and reorganization during spermiogenesis. The presence of multinucleated and flagellated spermatozoa in *DCUN1D1*^*-/-*^ mice suggests that DCUN1D1 affects individualization, a process similar to abscission in somatic cells. A unique variation in cytokinesis occurs during gametogenesis. Instead of undergoing abscission, the daughter cells develop as a syncytium, with clonally related cells connected by intercellular bridges [27, 28]. These intercellular bridges are evolutionarily conserved from invertebrates to humans and are removed during spermiogenesis, the final stage of spermatogenesis. Consistent with this, time-dam experiments mapped the defect in spermatogenesis in *DCUN1D1*^*-/-*^ mice to spermiogenesis. The expression of DCUN1D1 peaks during spermiogenesis and is associated with a corresponding increase in neddylated Cul3. Although previous studies have shown that Cul3^KLHL10^ plays a role in individualization in flies [29], the protein targets and substrate adaptors of Cul3 that are involved in individualization remain to be determined.

Why is the defect in abscission restricted to male germ cells? Previous studies have shown that mutations in genes that prevent formation or removal of intercellular bridges allow normal completion of mitosis and meiosis but result in sterility, with abnormal removal of intercellular bridges resulting in the development of multinucleated spermatids, owing to defective individualization [27, 30, 31]. However, genes that are essential for cytokinesis, formation of ring canals, or individualization in spermatogenesis are not universally required for faithful completion of oogenesis [32]. Interestingly, knockout of DCUN1D1 in *Drosophila* results in partial female infertility, likely reflecting similarities between spermatogenesis in mice and oogenesis in flies [17].

DCUN1D1-promoted neddylation of cullins and individual substrate adaptors cooperatively regulate assembly and subcellular location to provide tight control of CRL activity. As both DCUN1D1-promoted neddylation and substrate adaptors are involved, the functional contribution of DCUN1D1 to cellular activity needs to be assessed in the appropriate context. Our findings raise the question of how DCUN1D1 and its paralogs differentially regulate neddylation of the cullin family of proteins. DCUN1D1 is amplified and overexpressed in a variety of human cancers. We have previously shown that overexpression of DCUN1D1 activates its oncogenic activity *in vitro* and *in vivo*.

The increased proliferation resulting from DCUN1D1 overexpression in cell and animal models [10, 17], combined with the high prevalence of polyploidy in tumors with DCUN1D1 amplification, suggests the possibility that DCUN1D1’s cancer-promoting activity may result from its effects on abscission. Moreover, although proteasome and neddylation inhibitors have been shown to have therapeutic efficacy in humans, their broad-based activity induces severe side effects in a significant number of patients. The selective effects of DCUN1D1 in neddylation, combined with an “oncogene addiction” phenotype associated with its overexpression in human cancers, suggest that DCUN1D1 may be an excellent therapeutic target.

Our findings highlight the essential regulatory role of DCUN1D1 in neddylation in mammals, suggest the presence of functional redundancy in DCUN1D1 paralogs, and identify a protein target whose ubiquitination is controlled by DCUN1D1-mediated neddylation of CRLs. However, the factors regulating DCUN1D1 activity remain ill-defined. Moreover, it is also unclear how neddylation is coordinated with the availability of substrates primed for ubiquitination.

### Disclaimer

This article was prepared while Gary R. Hunnicutt was employed at the Population Council. The opinions expressed in this article are the author’s own and do not reflect the view of the National Institutes of Health, the Department of Health and Human Services, or the United States government.

## Supporting information

S1 Movie Spermatozoa from *DCUN1D1*^*+/+*^ mice have normal motility. For motility analysis, sperm were collected and analyzed as described in Experimental Procedures. Normal motility is seen in spermatozoa from *DCUN1D1*^*+/+*^ mice.
(MP4)

S2 Movie Spermatozoa from *DCUN1D1*^*-/-*^ mice have limited motility. For motility analysis, sperm were collected and analyzed as described in Experimental Procedures. Limited motility is seen only in morphologically abnormal, multiflagellated spermatozoa from *DCUN1D1*^*-/-*^ mice.
(MP4)
